# Comparison of Melatonin Profile and Alertness of Firefighters with Different Work Schedules

**DOI:** 10.5334/jcr.155

**Published:** 2018-02-21

**Authors:** Reza Kazemi, Sajad Zare, Rasoul Hemmatjo

**Affiliations:** 1 Department of Ergonomics, School of Health, Shiraz University of Medical Sciences, IR; 2 Department of Occupational Health, School of Health, Kerman University of Medical Sciences, Kerman, IR; 3 Department of Occupational Health, School of Health, Urmia University of Medical Sciences, Urmia, IR

**Keywords:** Night work, Melatonin, Sleepiness, Petrochemical firefighters

## Abstract

**Introduction:**

A two-shift work schedule with different rotations is common among firefighters in Iranian petrochemical companies. This study compared salivary melatonin and sleepiness on the last night before turning to day shift at 19:00, 23:00, 3:00, and 7:00 among petrochemical firefighters (PFFs) working seven and four consecutive night shifts.

**Methods:**

Sixty four PFFs working in the petrochemical industry were selected. To measure melatonin, saliva samples were taken, whereas the KSS index was used to assess sleepiness. Chi-square and independent samples t-test were carried out to analyze the data, and generalized linear model (GLM) was employed to determine the effect of confounding factors such as lighting and caffeine.

**Results:**

The levels of melatonin at 3:00 and 7:00, and the overall changes during the shift in the two shift patterns under the study were different (P < 0.05). Sleepiness was significantly different only at 3:00 in the two studied shift patterns, while the effects of lighting and caffeine on melatonin changes were not significant (P > 0.05).

**Conclusion:**

It seems that a slow shift rotation is better because it reduces the secretion of melatonin (hence reducing sleepiness during the night) and changes the peak of melatonin secretion to the daytime, which is a sign of adaptation.

## Introduction

As a growing type of work schedule, shift work is inevitable in many industries such as the petrochemical industry, steel industry, power plants, and essential services like police and hospital crew [1,2]. However, decreased alertness and performance, especially in night shifts, and inadequacy of daily sleep are considered fundamental problems [3,4]. The problems are due to the lack of synchronization between the internal circadian rhythm of the body and the sleep pattern and also the forced sleeplessness caused by the working conditions [3,5], which could lead to accidents or reduce the efficiency of the staff [6,7].

Although adaptation to night work (changes in the circadian phase) may have disadvantages, such as adverse effects on health and social life, adaptation to night work in shift workers is a proper strategy for increasing awareness during the night and improving sleep during the day [8]. Industries and the service sector use different shift patterns which differ in terms of length of the shift, type of the shift (fixed or rotating), speed of rotation, and the direction of shift rotation. Each shift pattern has its own advantages and disadvantages [3].

The speed of shift rotation is believed to be an important factor influencing people’s adaptation with the night work. It has been stated that the increase in the number of consecutive night shifts leads to changes in the circadian phase from night to day and improves people’s performance and alertness at night [3]. In one study, it was demonstrated that the adaptation to night work (change of circadian phase from night to day) occurs faster in the shift patterns with slow rotation (with seven or more consecutive night shifts) than in ones with quick rotation [9]. In addition, a comparison between the shift patterns with two, three, and four consecutive night shifts showed that people who worked four consecutive nights had better performance and were more alert than those who were working in two and three consecutive night shifts [9,10]. On the other hand, studies have indicated that larger number of consecutive night shifts enhances the likelihood of accidents and reduces cognitive performance [11].

The rhythm of melatonin and sleepiness, which is one of the valid and reliable markers for the assessment of adaptation with shift work patterns, has been addressed in several studies [8,12]. Melatonin is a hormone mainly secreted by the pineal gland and is a valid indicator of circadian rhythm; moreover, many biological cycles, including sleepiness, wakefulness, and alertness, are dependent on its changes. One of the reliable methods to measure melatonin in the body is through testing saliva, a noninvasive and simple method being applied by a growing number of researchers [13].

In shift work patterns, when the peak of melatonin secretion (acrophase) transfers to day sleep, it is said that the adaptation with night work occurs. In non-shift workers, concentration of melatonin decreases in the day and goes up at night. The characteristic of a normal rhythm of melatonin indicates that plasma melatonin reaches its minimum levels at 3 to 5 p.m. and its maximum level at 3 to 5 a.m. [14].

Although full adaptation is difficult due to the inertia of circadian rhythms, some studies have suggested that it is achievable after four to six nights, whereas some other studies have deemed it necessary to have a minimum of seven nights to achieve adaptation [4]. In a study by Barnes et al., which was conducted on offshore workers involved in two week night shifts and two week day shifts, it was reported that the delay in melatonin occurred during the first week of night work [15]. In addition to shift patterns, adaptation to night work depends on environmental variables such as lighting, individual differences, and seasonal factors [16].

Rates of adaptation with night work increase at higher light intensities [17]. Hansen et al. measured the urinary melatonin in offshore night workers and reported that the rate of phase change at each shift in an ambient light of 3 to 243 lux was 0.84 hour [16]; however, Barnes et al. demonstrated that the rate of phase change of melatonin in a light of 50 to 2000 lux in an oil platform was 1.77 hours [15]. Dumont et al. measured urinary melatonin at intervals of two hours in a day and three consecutive nights and reported that personal differences are effective in adaptation to night work. In their study, five participants showed a delayed phase change, three people displayed early phase change, and the remaining 22 subjects did not show any phase change (compared with fixed day workers) [17].

Studies on subjective and objective sleepiness have also shown that improvement in sleepiness index is associated with increased number of consecutive night shifts. More specifically, as the number of consecutive night shifts increases, sleepiness declines [3,17,18,19,20]. Waage et al. focused on comparing three shift work patterns of fixed day work, fixed night work, and rotating shift work among 19 offshore oil workers, with the results revealing that the highest scores of subjective and objective sleepiness were reported on the first day of working in fixed night work and rotating shifts, but the indicators of sleepiness improved over time [20].

Although many studies have evaluated melatonin secretion patterns and indicators of sleepiness in shift systems, few studies have compared the shift pattern with weekly rotation and less than a week rotations, which are common shift patterns in the petrochemical industry in Iran. On the other hand, working as a firefighter of petrochemical industries is considered to be very sensitive and requires operators’ alertness and good performance during the night work [21]. Therefore, it is very important to choose the most suitable shift system which helps the alertness and performance of the operators.

The aim of this study was to compare the salivary melatonin and sleepiness index of people working in the control room as firefighters at night under different night work patterns. The most appropriate shift patterns for the job (control room operation) are the ones that lead to reduced level of melatonin secretion at night and transfer its peak to the end of the shift so that people would become less sleepy during their work.

## Protocol of the study

In this study, we recruited a total of 80 individuals who were working as firefighters in a petrochemical company that is the largest petrochemical complex in south of Iran; they were equally distributed in two common shift work systems, including 7 night, 7 day and 7 off (7 N7D7O) and 4 N4D4O.

Given the aim of the study, which was to compare the two abovementioned models with emphasis on the impact of speed of rotation shifts on the pattern of melatonin secretion and sleepiness during the work, measurements were carried out on the last night before rotating shifts (on the seventh night (NNNNNNN) in the 7 N7D7O pattern, and on the fourth night (NNNN) in 4 N4D4O pattern. At the beginning of the study, a questionnaire was administered to collect participants’ demographic data. In this study the comparison was only between shift patterns — no assessment of differences between work days and non-work days was conducted.

To measure melatonin, four saliva samples were taken form each participant at four-hour intervals and the participants were also asked to record their subjective assessments of sleepiness at four-hour intervals.

Additionally, in order to control the influence of rest and schedule as well as duration and quality of sleep in day time, the participating workers were requested to avoid taking naps before the study and to have regular sleep schedules during both shift works and off days. It should be noted that, since staff members were living in camps constructed by the company far from their family, they all followed a relatively similar sleep-wake schedule from 8 a.m. to 3 p.m.

This study was conducted in accordance with the ethics guidelines established by Shiraz University of Medical Sciences. All participants provided informed consent prior to the experiment.

## Material and Methods

### Melatonin

Melatonin has been frequently used in previous studies as a very reliable indicator for circadian rhythm. In this study, salivary melatonin, which constitutes approximately 30% of plasma melatonin and is a reliable indicator, was measured. A growing number of studies are collecting samples from salivary melatonin because of the non-invasive nature of data collection. During the night shift, the samples were collected every four hours at 19:00–23:00–3:00–5:00–7:00 via a saliva sample collector (Sartsert, Germany). To limit the effects of food on melatonin, subjects were asked to avoid eating one hour before sampling. The collected samples were immediately centrifuged, frozen, and stored at –20°C and were subsequently transferred to the laboratory. An ELISA kit (manufactured by Biotech Company in China) was used to measure melatonin levels. The sensitivity of the tests was 1.6 ± 1.3 pg/ml. Intra-assay coefficient of variation was 8.1% at 1.8 pg/ml and 5.5% at 25 pg/ml.

### Sleepiness Scale (Karolinska Sleepiness Scale/KSS)

In this study, to assess subjective sleepiness Karolinska Sleepiness Scale (KSS) was used. The instrument measures participants’ sleepiness on a nine-point Likert scale with anchors at 1 = very alert, 3 = alert, 5 = neither alert and nor sleepy, 7 = sleepy, and 9 = very sleepy and trying to stay awake. Previous research has indicated that KSS is fairly reliable and valid for measuring individuals’ sleepiness [22]. In this study, each participant recorded its own sleepiness level four times every four hours.

### Exposure to light

Three times during the night shift, light exposure was measured using a lux meter (Extech Light Meter LT300, USA). It measured light exposure from 0 to 100,000 lux, at eye level in the center of the work area. Light exposure measurements were simultaneous with saliva and sleepiness assessments.

### Statistical analysis

The collected data were analyzed using SPSS Version 18. The univariate analysis was carried out via chi-square and independent samples t-test. In addition, repeated measurement analysis of variance was used to control the effect of within subjects, light, and caffeine. In the analysis, light and caffeine were selected as the covariates and the trend of melatonin and sleepiness changes were used as dependent variables. To calculate the overall changes in melatonin and sleepiness, the fourth measured value was subtracted from the first measured value. In our analysis, 0.05 was determined as the significance level.

## Results

The mean ± sd age of participants was 32.16 ± 2.5 years and they had 4.5 ± 1.4 years of shift work experience. As reported by the subjects, the mean ± sd subjective sleep quantity and quality of the studied subjects were 6.7 ± 1.04 hours and 6.5 ± 3.4, respectively. Overall, 63.3% had Bachelor of Science or higher educational degrees and the rest of the participants held Associate Diploma. The mean ± sd of caffeine cups consumed per shift was 2.79 ± 1.33 and the mean of light exposure in work place was calculated as 542.7 ± 38.8 lux.

Table 1 shows that there was no difference between the two groups in terms of education level, sleep quantity and quality, shift work experience, age, and caffeine consumption. Nonetheless, a significant difference was observed in light exposure.

**Table 1:** Comparison of characteristics and baseline variables between the two night shifts.

Variables	7-night shift (slow rotation)	4-night shift (fast rotation)	P value

Age (year)	31.10 ± 2.60	29.20 ± 1.90	**0.003‡**
Shift work experience (year)	6.30 ± 1.00	5.50 ± .90	**0.060‡**
Sleep quantity (h)	7.00 ± 1.12	6.47 ± 0.95	0.267‡
Sleep quality	6.70 ± 3.62	6.40 ± 3.12	0.163‡
Light (lx)	530.50 ± 34.20	554.90 ± 39.80	**0.013‡**
Caffeinated drinks (cup)	2.72 ± 1.46	2.86 ± 1.20	0.698‡
BSc or higher degree n(%)	19.00(63.30)	19.00(63.30)	0.605†

‡ Independent-t-test, † χ2.

As illustrated in Table 2, the independent samples t-test showed no significant difference between the first and second measurements of melatonin between the two shifts. Nevertheless, the value of melatonin in the third and fourth measurements and the overall change of melatonin was statistically different between the two shifts (p < 0.05). Moreover, the difference in the level of sleepiness between the two groups was not statistically significant in any measurement (p > 0.05), except for the third one.

**Table 2:** Comparison of melatonin and sleepiness between the two night shifts.

Variables	7-night shift	4-night shift	P value

melatonin 1	5.71 ± 3.00	6.21 ± 2.80	0.503‡
melatonin 2	10.61 ± 5.62	8.67 ± 4.00	0.103‡
melatonin 3	16.24 ± 6.76	26.35 ± 9.81	**<0.001‡**
melatonin 4	21.57 ± 7.92	15.07 ± 5.80	**<0.001**
Overall Melatonin changes	17.3 ± 7.90	9.77 ± 5.70	**<0.001**
Sleepiness 1	2.86 ± 1.09	2.48 ± 0.57	0.104‡
Sleepiness 2	3.13 ± 1.17	1.23 ± 1.36	0.684‡
Sleepiness 3	4.30 ± 1.15	6.63 ± 1.59	**0.049‡**
Sleepiness 4	6.50 ± 2.39	5.60 ± 2.01	0.273‡
Sleepiness changes	4.62 ± 2.54	4.14 ± 2.2	0.442

**Figure 1:**
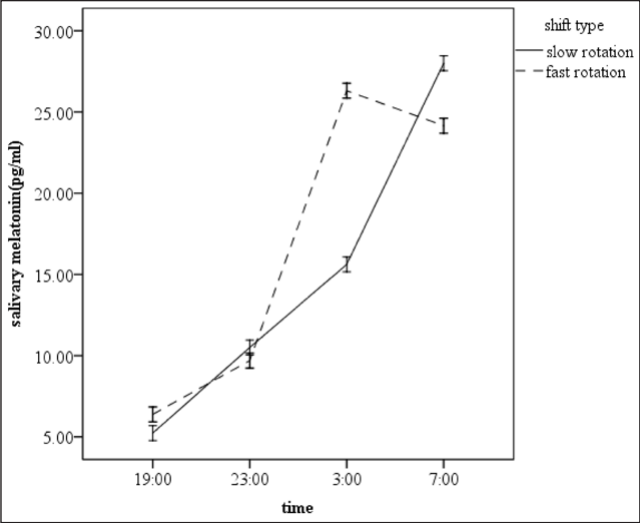
The trend of melatonin changes for each shift.

**Figure 2:**
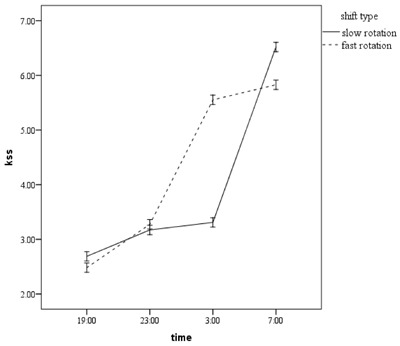
The trend of sleepiness changes for each shift.

The repeated measurement analysis of variance showed that there was a significant difference between subjects in the two types of shift in the four measurements of melatonin (p < 0.001). Moreover, the overall trend of changes in melatonin after controlling for light and caffeine were statistically significant between the two shifts (p < 0.001). Figure 1 shows the trend of increasing melatonin in each shift. In addition, there was a significant increase in the trend of sleepiness (Figure 2) in the four measurements and between the two shifts, after controlling for light and caffeine (p < 0.001). However, the interactive effect of light and caffeine on changes in melatonin and sleepiness was not statistically significant (P > 0.05). However, the interaction between light and sleepiness was very close to the significant level (p = 0.055).

## Discussion

In this field study, we sought to compare the rhythms of melatonin and sleepiness between people working in two different night shift systems; the study was conducted among firefighters working in the petrochemical industry. The overall analysis showed that the adaptation was better in the 7 N7D7O than in the 4 N4D4O shift pattern. There was a significant difference between the two groups in terms of night melatonin rhythm and the most important difference was observed in the peak of melatonin secretion. The change in melatonin acrophase (peak melatonin secretion) has been used in different studies as a reliable criterion to assess adaptation with the night work [14]. To measure the peak of melatonin rhythm it is required to measure its level during 24 hours a day. In this study, nonetheless, only the rhythm of melatonin at night was evaluated. However, the difference between peak melatonin formations at the night shift among the people working in the two studied shift patterns can be a good indicator of adaptation. The maximum level of melatonin in people working in 7 N7D7O shift pattern was detected at the end of the shift, while the maximum measured level of melatonin in 4 N4D4O shift pattern was observed at about 3:00 pm.

Considering that the peak of melatonin secretion in normal people (non-shift workers) forms at about 3:00 or 4:00 a.m. [13], it can be concluded that, at the end of the night shift period, people working in 7 N7D7O shift pattern have a better adaptation to night work than people working in 4 N4D4O shift pattern. These results corroborate the findings of Haidarimoghadam et al.’s study [3], which demonstrated that the cognitive performance of workers in the 7 N7D7O shift pattern is significantly better than that of people following the 4 N4D4O pattern. The main reason for this difference can be the discrepancy between the number of consecutive night shifts, which has also been echoed by the results of studies on offshore workers and experimental studies [23]. In a study conducted among offshore workers, the adaptation rate based on the changes in melatonin acrophase was about 1.5 h/shift, and the full adaptation occurred at the end of 14 consecutive nights [24]. In another study, it was shown that the adaptation to night work was achieved over 4 to 7 consecutive night shifts and melatonin acrophase changed from night to day [3]. Also, another study found that melatonin rhythm did not change in workers who were working in a quick rotating shift pattern. However, it is not consistent with many field studies which reported that adaptation to night work was impossible or very slow [25]. For example, Roeden et al. examined the melatonin rhythm of night workers and showed that in a large number of the subjects, as compared with day workers, the phase change in melatonin rhythm did not happen [26]. This inconsistency can be attributed to environmental conditions such as exposure to light, individual differences (e.g. being morning-oriented), and social/family commitments of the subjects in various studies [16,25]. Suppression of melatonin caused by exposure to morning light had been mentioned as a strong factor preventing the adaptation to night work. On the other hand, studies on people working offshore and simulated studies have shown that easier adaptation of people to night work stemmed from the lack of daily social and family commitments and obligations [27]. In addition, the peak of melatonin is formed earlier in morning-oriented people than in evening-oriented people, and they have more difficulty to adapt to night work [8].

In this study, given that some of the subjects were away from their families and their accommodation was close to their work, they were less likely to be exposed to the morning light. In addition, their social and family commitments during the day was negligible, so that they could adapt themselves to the conditions.

It should be noted that although the shift of melatonin peak from night to day can contribute to workers’ better adaptation to night shift work and improved quality and quantity of sleep, it may lead to some familial and social problems. For example, night shift workers will face some performance problems while they attend to their familial and social engagements during the day. In the present study, since the participants worked in a remote area and lived far from their families, they did not have any familial dependence. Thus, the abovementioned problem was not an issue for them.

Another variable in this study was the subjective sleepiness index. The results showed that the index of subjective sleepiness was different only at 3 a.m. and was significantly different between the two shift patterns. This finding corresponds with the amount of melatonin observed at this time. As mentioned earlier, the amounts of melatonin secretion at that time showed significant differences in both groups and reached its peak in workers under 4 N4D4O shift pattern. This finding also confirms the better adaptation of workers in 7 N7D7O than in 4 N4D4O shift pattern. The peak of night fatigue occurs at 3 a.m. [9] and the maximum amount of melatonin is secreted in normal people (non-shift workers) at this time [13]. Moreover, people in the shift pattern with four consecutive nights suffer more from sleepiness than in shift patterns with seven consecutive nights of work. It indicates that when the number of consecutive nights increases, the adaptation to night work goes up. The results of this study are consistent with the findings of Waage et al.’s study, which showed that with increasing the number of consecutive night shifts, sleepiness decreases due to the adaptation of circadian rhythm [20]. Moreover, in a study by SD Baulk, sleepiness declined in the second night as compared with the first night, which is in line with the results of our study [28]. The similarity between the values of sleepiness index at the beginning of a shift in both studied groups can be attributed to daily sleep before each night shift, which was not investigated in our study. Studies have shown that short sleep before night shifts prevent people from sleepiness during night work [29]. In addition, the similarity in sleepiness at the end of the shift in the two groups can be due to the sleep-related processes. Sleep is determined by two processes of circadian rhythm and homeostatic process of the mind. With regard to circadian rhythm, increasing the body’s level of melatonin leads to a rise in the sleepiness, resulting in full sleep. Concerning homeostatic process, as the time of wakefulness increases, homeostatic sleep goes up as well. Hence, given the similar duration of wakefulness during the work in both shift patterns, a similar level of sleepiness was observed at the end of the shift, which might be attributed to homeostatic sleep process.

## Limitations of the study

Like any other study, the current research suffers from some shortcomings:

1. The results of this study indicated that increasing the number of consecutive night shifts will lead to reduction in the level of melatonin and sleepiness, hence improving alertness. Nonetheless, previous research has revealed that melatonin suppression may have adverse effects on health in the long run. We measured melatonin only during night shifts, thus failing to assess the level of melatonin in both groups over an entire day. Future studies should collect samples in regular intervals during a 24-hour period to come up with a more accurate measurement of melatonin.
2. The current study only focused on the effect of shift work patterns on sleepiness and melatonin level without examining the influence of shift pattern on individuals’ health. Given that melatonin suppression can have negative effect on health, other studies should also examine the impact of shift patterns on workers’ health. By so doing, we will be able to find out the shift pattern that is more appropriate for keeping people healthy.
3. In this study, we limited ourselves to only one night shift in the entire work cycle. In order to determine the period of desynchronization in each group, future research should focus on all consecutive night shifts of the work cycle.
4. The last limitation has to do with the frequency of melatonin measurement and sleepiness during the shift. Due to the participants’ unwillingness, we could not conduct more than 4 measurements (in 4 hour intervals) during each shift. Having a larger number of measurements presents a better opportunity to assess changes in melatonin and sleepiness during the shift. It is therefore suggested that future researchers try to make a larger number of measurements.

## Conclusion

In general, it can be argued that the work shift system with seven consecutive night shifts leads to better adaptation to night work because it moves the melatonin acrophase to the end of the shift. In addition, in this shift pattern, the sleepiness index during the shift is lower than that in 4 N4D4O shift pattern. This will lead to better performance of workers in the 7 N7D7O schedule and may reduce human errors caused by sleepiness and fatigue. Hence, since people working in control rooms in the petrochemical industry need to be properly aware of their surroundings, it is recommended that decision makers avoid using fast rotating shift systems as they prevent shift workers from adapting to night shifts. However, it should be noted that the subjects employed for this study were working under particular conditions (especially, in terms of working type and lifestyle, e.g. workers lived far from their families). Therefore, caution must be taken in generalizing the findings to other contexts with different working conditions.
